# Prevalence and Associated Factors with Ideal Cardiovascular Health Metrics in Bangladesh: Analysis of the Nationally Representative STEPS 2018 Survey

**DOI:** 10.3390/epidemiologia3040040

**Published:** 2022-11-30

**Authors:** Rajat Das Gupta, Rownak Jahan Tamanna, Mohammad Rashidul Hashan, Maxwell Akonde, Shams Shabab Haider, Promit Ananyo Chakraborty, Md. Belal Hossain

**Affiliations:** 1 Department of Epidemiology and Biostatistics, Arnold School of Public Health, University of South Carolina, Columbia, SC 29208, USA; 2 Institute of Statistical Research and Training (ISRT), University of Dhaka, Dhaka 1000, Bangladesh; 3 Bangladesh Civil Service, Ministry of Health and Family Welfare, Government of Bangladesh, Dhaka 1000, Bangladesh; 4 School of Health, Medical and Applied Sciences, Central Queensland University, Rockhampton North, Queensland 4701, Australia; 5 Johns Hopkins Bloomberg School of Public Health, Johns Hopkins University, Baltimore, MD 21205, USA; 6 School of Population and Public Health, University of British Columbia, Vancouver, BC V6T 1Z8, Canada

**Keywords:** cardiovascular disease, heart disease risk factors, non-communicable diseases

## Abstract

This study aims to find out the prevalence of the American Heart Association’s (AHA)’s cardiovascular health metrics and associated socio-demographic factors. A secondary analysis of the World Health Organization (WHO) STEPwise approach to surveillance survey 2018 (STEPS 2018) data was conducted. Ideal Cardiovascular Health (ICH) was defined as the presence of 5–7 ideal metrics as defined by the AHA. Design-adjusted multivariable logistic regression was used to determine the associated factors of ICH. In total, 5930 respondents were included in our analysis, and 43.1% of the participants had ICH. The odds of ICH decreased with age [compared to 18–29 years old individuals, 30–49 years: AOR (Adjusted Odds Ratio): 0.4; 95% Confidence Interval (CI): 0.4–0.5; 50–69 years: AOR: 0.7; 95% CI: 0.6–0.8], and higher educational attainment (compared to those who received no formal education, being educated up to primary level: AOR:0.7; 95% CI: 0.6–0.8; being educated up to secondary level: AOR: 0.4; 95% CI: 0.4–0.5; being educated up to college and higher: AOR: 0.4; 95% CI: 0.3–0.5). Compared with female and urban residents, the odds were 30% and 40% less among male and rural residents, respectively. The public health promotion programs of Bangladesh should raise awareness among high-risk groups to prevent cardiovascular diseases.

## 1. Introduction

Cardiovascular diseases (CVDs) are one of the leading contributors to the non-communicable disease (NCD) burden, resulting in nearly one-third of global annual deaths [1]. The Global Burden of Disease 2019 study reported that the prevalence of total CVD cases almost doubled over the last two decades from 271 million (95% CI 257–285) to 523 (95% CI 497–550) million, with over three-quarters of mortality occurring mostly in low and middle-income countries [1,2]. Major CVDs can be prevented by addressing modifiable risk factors, such as smoking, high blood pressure, fasting blood glucose, physical activity, and blood cholesterol stringently, as highlighted in several clinical guidelines [3,4].

In 2010, as a response to the continued rising burden of CVDs worldwide and increased adoption of unhealthy sedentary lifestyles, the American Health Association (AHA) devised population-wide strategies to promote cardiovascular health [5]. The AHA developed a strategy underscoring primary prevention using the concept of ideal cardiovascular health (ICH) as determined by seven key metrics, which included certain health behaviors (smoking, physical activity, diet, and body mass index) and biological factors (blood pressure, fasting plasma glucose (FPG), and total cholesterol) [5]. According to earlier studies, meeting a higher number of the observed ICH metrics was found to be associated with substantially reduced risks of all-cause and CVDs mortality as well as incidence and prevalence of CVD-related events [6,7].

The prevalence of NCDs and associated mortality have been significantly higher in Bangladesh over the previous few decades [8]. Global burden of disease evidence showed CVDs as a leading cause of all-age mortality in Bangladesh, with an increase in the proportion of stroke and ischemic heart disease by 22.8% and 47.2%, respectively, over the last decade [8]. Very few studies have been conducted on the burden of CVDs on the Bangladeshi population; however, they have been conducted on specific populations (i.e., urban/rural, specific population) [9,10]. Due to the lack of systematic national population-based surveys or a central health database, it is a major public health challenge to measure the magnitude of cardiovascular health status in this demography. Moreover, ongoing urbanization and economic advancement have been identified as key drivers in the mounting NCD epidemic [11]. Several studies from low and middle-income countries reported multiple socio-demographic factors such as age, sex, educational status, region of residence, and income status as determinants of population-level cardiovascular health metrics [12,13,14]. Therefore, it is crucially important to identify these socio-demographic groups and factors that can offer potential knowledge to formulate policies and establish guidelines to mitigate such alarming CVD burden in the context of Bangladesh.

To our knowledge, AHA-proposed ICH metrics have not yet been explored broadly in the setting of the ongoing epidemiologic transition toward a rising CVD burden within Bangladesh. To develop effective preventive approaches, it would be of substantial importance to measure cardiovascular health metrics across the population level. In this study, we aim to investigate the prevalence of the AHA’s cardiovascular health metrics and associated socio-demographic factors utilizing nationally representative data.

## 2. Materials and Methods

### 2.1. Study Design, Setting, and Sampling

A secondary analysis of the 2018 World Health Organization (WHO) STEPwise approach to surveillance (STEPS 2018) survey data was conducted. The STEPS 2018 was a nationally representative survey (cross-sectional study) conducted in Bangladesh from February to May 2018. The aim of this survey was to generate the latest updated information on different indicators related to NCDs. The survey methods, sampling strategy, data collection tools, and data collection methods were published previously [15,16].

In brief, a two-staged stratified cluster sampling technique was followed [15]. The survey utilized the sampling frame of the Population and Housing Census 2011 developed by the Bangladesh Bureau of Statistics. In total, 496 primary sampling units (PSUs) were selected from all over Bangladesh. An equal number of PSUs were chosen from both urban and rural areas (*n* = 248) and from all eight administrative divisions of Bangladesh (*n* = 62; urban *n* = 31 and rural *n* = 31, from each division). One PSU was excluded due to inaccessibility. In total, 495 PSUs were selected for the final survey [15]. Through systematic random sampling, a fixed number of households (*n* = 20) were selected from each PSU. Adults aged 18–69 years, living in the selected households for a minimum duration of six months, and being present at the time of the survey were eligible to participate in the survey. One individual was randomly selected for the survey from each household. Exclusion criteria were (1) being a resident of prisons, hospitals, nursing homes, and military barracks, (2) suffering from frailty/physical unfitness, and (3) being unable/unwilling to participate in the survey.

### 2.2. Data Collection

A pre-tested standard questionnaire was used for data collection. The questionnaire was developed following the WHO STEPS questionnaire (V.3.2) [15]. The original questionnaire was in English. It was translated into Bengali, then back-translated into English.

Trained data enumerators collected the data under field supervision. At first (STEP-1), face-to-face interviews were conducted to collect data on the background characteristics of the respondents, as well as their behavioral risk factors related to diet, physical activity, and tobacco and alcohol use. In the second step (STEP-2), physical measurements were taken. These included height (measured by a portable stadiometer), weight (measured by a digital weight measuring machine), waist circumference (measured by measuring tape), and blood pressure measurement (measured by WHO supplied digital sphygmomanometer with uniform cuff) [15,16]. In the final step (STEP-3), blood and urine were collected under aseptic precautions [15,16]. During the date of data collection (STEP-1 and STEP-2), the participants were given a card that contained the appointment date, time, and place for the serum total cholesterol and serum FPG. Participants were instructed to fast overnight for 12 h. Diabetic patients were instructed to bring their medication with them and take the medication after providing a blood sample. On the date of the appointment, serum total cholesterol and serum FPG was measured using a cardio-check [15].

### 2.3. Study Variables

The outcome of interest of this study was cardiovascular health status, which was defined on the basis of the presence of the following ideal health metrics: blood pressure (BP), serum total cholesterol (TC), serum fasting plasma glucose (FPG), smoking behavior, body mass index (BMI), physical activity, and an ideal diet.

(1) Blood pressure (BP): Untreated systolic blood pressure (SBP) < 120 mmHg and diastolic blood pressure (DBP) < 80 mmHg [5].
(2) Serum total cholesterol (TC): <200 mg/dL without any cholesterol-lowering medications [5].
(3) Serum fasting plasma glucose (FPG): <100 mg/dL without any glucose-lowering medications [5].
(4) Smoking behavior: Never smoke [5].
(5) Body Mass Index (BMI): Ideal BMI (BMI = 18.5–<25 kg/m^2^) [5].
(6) Physical activity: Adequate physical activity was defined as engaging in at least 300 min of moderate-intensity aerobic physical activity per week or at least 150 min of vigorous-intensity aerobic physical activity per week [5,17].
(7) Ideal Diet/Adequate fruit and vegetable intake: Adequate fruit and vegetable intake was defined as an intake of at least 4.5 servings a day [5,15].

Cardiovascular health was categorized as the following: (1) ideal cardiovascular health (ICH; 5–7 ideal metrics); (2) intermediate cardiovascular health (3–4 ideal metrics); (3) poor cardiovascular health (0–2 ideal metrics) [18]. The definition of the cardiovascular health metrics is presented in Supplementary Table S1.

The following independent variables were considered based on the literature review and availability in the STEPS 2018 dataset [18]: age group (18–29 years, 30–49 years, 50–69 years), sex (female, male), education (no formal education, primary, secondary, college and higher), marital status (never married, currently married, divorced/widowed/separated), place of residence (urban, rural), division of residence (Dhaka Rural, Barisal, Chittagong, Khulna, Mymensingh, Rajshahi, Rangpur, Sylhet), religion (Islam, Others). In the ‘others’ category of religion, participants following Hinduism, Christianity, and Buddhism were merged into a single category.

Although the name of the subcategory in the division of residence variable is ‘Dhaka Rural’, it contained respondents from both urban and rural areas (Supplementary Table S2).

### 2.4. Statistical Analysis

All statistical analyses were conducted using STATA version 16.0 (College Station, TX, USA). At first, descriptive analysis was conducted, and the findings were reported in unweighted frequency with weighted proportions. The sampling weight of WHO STEPS was used during the analyses. Since the proportion of missing data was less than 2.5% and the sample size was large, we performed a complete case analysis [19]. In order to find the differences among the groups, the Rao-Scott Chi-square test was utilized. Design-adjusted logistic regression analyses were conducted to determine the factors associated with the ideal cardiovascular metric. At first, the outcome variable was dichotomized into two categories: intermediate and poor cardiovascular health were merged into one category, while ideal cardiovascular health was considered as one category. Then, the combined intermediate/poor cardiovascular health category was considered as the base for the ICH odds calculation. At first, the crude odds ratio (COR) was calculated through bivariate analysis. Then multivariable logistic regression was conducted to identify the adjusted odds ratio (AOR). A confidence interval (CI) of 95% was reported, and a *p*-value of <0.05 was considered to be statistically significant. The presence of multicollinearity was checked by the variance inflation factor (VIF). A VIF cut-off of five was considered to be an indication of multicollinearity [20]. However, no multicollinearity was reported. The estimated VIF was 1.71.

### 2.5. Ethical Considerations

The Bangladesh Medical Research Council approved the protocol of STEPS 2018 [15]. Before data collection, written informed consent was collected from the respondents [15]. In April 2021, electronic permission was obtained from the NCD Microdata Repository of WHO for using the dataset in the current research. This study was exempted from review by the Institutional Review Board of the respective institutes due to the utilization of de-identified data.

## 3. Results

### 3.1. Background Characteristics of the Participants

The background characteristics of the participants are presented in Table 1.

In total, 5930 respondents were included in our analysis. Only 21.8% of the respondents were aged between 50–69 years. A majority of the respondents (78.4%) either had no formal education or were educated up to primary level. A majority of the participants were from rural areas (78.9%).

### 3.2. Prevalence of ICH by Background Characteristics

The prevalence of ICH by background characteristics is shown in Table 1.

In total, 43.1% of respondents had overall ICH. The prevalence of ICH decreased with increasing age (*p* < 0.0001) and increased educational attainment (*p* < 0.0001). Males had a lower prevalence of ICH compared to their female counterparts (Male vs. Female: 38.0%; 95% CI: 35.1%, 41.0% vs. 47.3%; 95% CI: 44.5%–50.1%; *p* < 0.0001). Urban residents had a lower prevalence of ICH compared to their rural counterparts (Urban vs. Rural: 34.2%; 95% CI: 31.4%–37.1% vs. 45.5%; 95% CI: 44.5%–50.1%; *p* < 0.0001). Among the places of residence, participants from Rangpur Division had the highest prevalence (60.0%; 95% CI: 54.2%; 65.6%), and participants from Khulna Division had the lowest prevalence of ICH (31.3%; 95% CI: 26.8%, 36.2%). Although not statistically significant (*p* = 0.3226), participants following Islam (43.7%; 95% CI: 41.4%, 45.9%) had a higher prevalence of ICH compared to participants following other religions (39.1%; 95% CI: 33.1%, 45.5%). Participants who were never married also had a higher prevalence of ICH compared to those who were currently married or ‘divorced/widowed/separated’ (*p* < 0.001).

**Figure 1:**
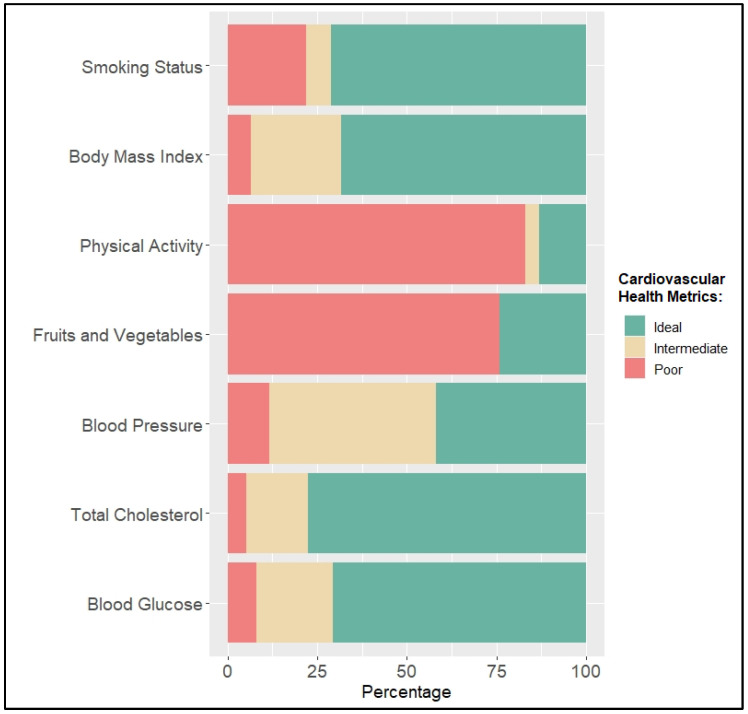
Prevalence of poor, intermediate, and ICH metrics for each of the seven cardiovascular health metrics.

The distribution of poor, intermediate, and ideal cardiovascular health categories for each of the seven AHA cardiovascular health metrics components is shown in Figure 1. Among the participants, ideal blood cholesterol was most prevalent (77.5%), and ideal physical activity (13.2%) was least prevalent.

### 3.3. Factors Associated with ICH:

Table 2 presents the factors associated with ICH among the adult Bangladeshi population. After adjusting for all the covariates, the following factors were found to be significantly associated with ICH: age, sex, education, marital status, place of residence, and division of residence.

The odds of ICH decreased with increasing age. Participants aged 30–49 years and 50–69 years had 60% (AOR: 0.4; 95% CI: 0.4–0.5) and 80% (AOR: 0.2; 95% CI: 0.2–0.3) lower odds of having ICH compared to individuals aged 18–29 years. The odds were 30% less among males than females (AOR: 0.7; 95% CI: 0.6–0.8).

Increased educational status was associated with decreased odds of having ICH. Compared to participants who had no formal education, people who received education up to primary level had 30% lower odds of ICH (AOR: 0.7; 95% CI: 0.6–0.8). For the individuals being educated up to secondary level and ‘college and higher’, the odds were 60% less than those with no formal education (secondary: AOR: 0.4; 95% CI: 0.4–0.5; college and higher: AOR: 0.4; 95% CI: 0.3–0.5).

The odds of having ICH were 50% and 60% less for those who were currently married (AOR: 0.5; 95% CI: 0.4–0.7) and divorced/widowed/separated (AOR: 0.4; 95% CI: 0.3–0.6), respectively, compared to the respondents who were never married.

Urban residents had 40% lower odds of achieving ICH compared to rural residents (AOR: 0.6; 95% CI: 0.6–0.7).

Division of residence was also associated with ICH. Compared to the residents of Dhaka Rural, the odds were 30% higher among the residents of Chittagong Division (AOR: 1.3; 95% CI: 1.0–1.6) and 60% higher among the residents of Mymensingh Division (AOR: 1.6; 95% CI: 1.2–1.9). Meanwhile, the odds were doubled among the residents of Rangpur Division (AOR: 2.0; 95% CI: 1.6–2.5). Finally, the odds were 30% less among the residents of Khulna Division (AOR: 0.7; 95% CI: 0.6–0.9).

## 4. Discussion

In this study, we aimed to find the prevalence of AHA-recommended cardiovascular health metrics and associated socio-demographic factors utilizing the nationally representative STEPS 2018 data. Three in every five participants had an intermediate to poor cardiovascular health metric score (i.e., achieving < 5 of the 7 ICH metrics). Age, sex, education status, marital status, and place and division of residence were found to be associated with one’s ICH metric.

In this study, 43.1% of respondents had overall ICH (five to seven ideal metrics). This is lower than Nepal (51.6%) but higher than Peru (12.7%) [18,21]. This difference might be due to the difference in study population, setting, and methodology used in determining the prevalence of cardiovascular health metrics.

In this study, we found that the prevalence of ICH metrics was less than 50% in several components. For example, only 13.21% of participants followed ideal physical activity. Regular physical activity reduces the risk of CVD. Wahid et al. (2016), in their systematic review and meta-analysis, showed that having 150 min of moderate-intensity aerobic activity per week is associated with a 17% reduction in cardiovascular disease risk and a 23% reduction in the risk of cardiovascular disease mortality [22]. Only 24.19% of respondents reached the daily recommended intake of fruits and vegetables. Fruit and vegetable intake of 200 g per day is associated with an 8% reduction in the risk of coronary heart disease and CVD mortality [23]. Approximately three-fifths of the individuals did not have ideal blood pressure. Around 11.69% of them did not have their blood pressure controlled. Uncontrolled hypertension significantly increases the risk of mortality due to cardiovascular disease [24]. In addition, 21.91% of individuals were active smokers, which increases their risk of cardiovascular disease [25].

We found that the odds of attaining ICH decreased with age. Adults aged 50–69 years had 80% lower odds of having ICH compared to individuals aged 18–29 years. Similar findings were reported in Nepal [18]. Age is a non-modifiable factor for cardiovascular disease [26]. With increasing age, the risk of hypertension, diabetes, and high cholesterol increases [26,27,28]. As such, with increasing age, the probability of maintaining ICH decreases.

Women had a higher probability of achieving ICH than men. The majority of our participants (approximately 65%) were aged equal to or less than 45 years. Among this age group, the prevalence of cardiovascular disease risk factors (i.e., hypertension and high blood cholesterol) are higher among males. This might explain the higher probability of women having ICH compared to men within our study population. Prior to menopausal age, estrogen plays a cardioprotective role among females [29]. After menopause, the risk increases among women [30].

The odds of achieving ICH decreased with increased educational attainment. In low-and-middle-income countries, people with higher educational attainment are usually involved in sedentary activities. As a result, the risk factors for cardiovascular disease (i.e., hypertension, high BMI, high blood sugar, and high cholesterol level) are highly prevalent among this population [31].

The probability of achieving ICH was higher among rural respondents compared to their urban counterparts. Residents of rural areas are more prone to engaging in physical activity compared to urban residents [32]. As a result, the prevalence of risk factors for cardiovascular disease is lower among rural residents. Public health promotion programs in Bangladesh should raise awareness among elderly individuals, males, and individuals with higher educational attainment.

Marital status was also associated with cardiovascular health. Currently, married and divorced/widowed/separated respondents were less likely to have ICH compared to never-married respondents. This is contrary to the systematic review and meta-analysis conducted by Wong et al. (2018) [33]. In that study, the authors found that compared to currently married individuals, single (never married) and divorced/widowed/separated individuals had a higher risk of having cardiovascular disease [33]. This might be due to differences in the context of the study. Wong et al.’s study involved the populations of mainly upper-income nations. Further research is necessary to understand why the probability of ICH is lower among currently married and divorced/widowed/separated respondents.

There were also regional differences in the odds of ICH. Compared to the residents of Dhaka rural, the odds of ICH were higher for the residents of the Chittagong, Mymensingh, and Rangpur divisions, and the odds were lower for the Khulna division [34]. This might be due to differences in lifestyle and other factors. For instance, in the Khulna division, there is salinity in drinking water, which predisposes the population towards cardiovascular disease and hypertension. Further research is needed in order to explore the reason for these regional differences.

The Government of Bangladesh recently adopted a multi-sectoral action plan for the prevention and control of non-communicable diseases [35]. This multi-sectoral action plan aims to reduce premature mortality from non-communicable diseases by 25% by 2025 [34]. It also aims to increase health promotion and risk reduction activities [34]. The health promotion programs can tailor their interventions according to high-risk groups. Also, implementation research should be incorporated within the programs in order to understand what works and what does not work.

This study has several strengths. First, it used a nationally representative sample. As a result, the findings of this study can be generalizable to the target population. Second, STEPS 2018 utilized validated tools for data collection and calibrated tools for measurement [15]. As a result, the probability of measurement error and information bias is limited. However, there are several limitations. This is a cross-sectional study, which impedes our ability to make causal inferences. The STEPS 2018 dataset did not have information on household wealth status, restricting our ability to include the variable in the analysis. There also may be an issue of recall bias. Finally, the survey was not planned to estimate ICH, and thus, we may need to interpret the group-specific prevalence with caution.

## 5. Conclusions

This study found that approximately three-fifths of Bangladeshi adults aged 18–69 years fell short of ideal cardiovascular health. Increasing age, male gender, increased educational attainment, the status of currently married and divorced/widowed/separated, urban residence, and residence within the Khulna division were all factors indicating a lower probability of attaining ICH. Public health promotion programs in Bangladesh aiming to prevent and control cardiovascular disease should focus on these high-risk groups. Further research is required to understand the impact of wealth status on ICH. The influence of marital status and division of residence should also be explored.

## Figures and Tables

**Table 1:** Background characteristics of the participants according to cardiovascular health metrics (*N* = 5930).

Background Characteristics	Total Population	Ideal Cardiovascular Health (5–7 Ideal Metrics)	Intermediate Cardiovascular Health (3–4 Ideal Metrics)	Poor Cardiovascular Health (0–2 Ideal Metrics)	*p* Value
	*n* (%)	%	%	%	
Age Group (in years)					<0.0001
18–29	1244 (27.1)	58.0 (54.0–61.9)	36.4 (32.6–40.4)	5.6 (4.1–7.5)	
30–49	3371 (51.1)	40.9 (38.2–43.7)	45.7 (43.2–48.3)	13.4 (11.7–15.4)	
50–69	1315 (21.8)	29.6 (26.0–33.5)	54.4 (50.6–58.2)	16.0 (13.6–18.7)	
Sex					<0.0001
Female	3209 (54.7)	47.3 (44.5–50.1)	43.8 (41.2–46.4)	9.0 (7.6–10.5)	
Male	2721 (45.3)	38.0 (35.1–41.0)	46.7 (44.2–49.3)	15.3 (13.5–17.4)	
Education					0.0053
No formal	1752 (31.5)	45.5 (42.1–48.9)	44.6 (41.6–47.6)	9.9 (8.1–12.1)	
Primary	2762 (46.9)	43.9 (40.8–47.0)	44.8 (42.1–47.5)	11.3 (9.8–13.1)	
Secondary	1009 (16.5)	39.1 (34.7–43.7)	45.8 (41.2–50.4)	15.2 (12.1–18.9)	
College and Higher	407 (5.1)	33.8 (26.6–41.8)	48.7 (41.0–56.4)	17.6 (12.6–24.0)	
Marital Status					<0.0001
Never married	339 (7.4)	61.6 (54.4–68.4)	34.8 (28.2–42.1)	3.6 (1.8–7.0)	
Currently married	5313 (88.4)	42.1 (39.9–44.4)	45.5 (43.6–47.5)	12.4 (11.1–13.8)	
Divorced/widowed/separated	278 (4.2)	30.0 (22.8–38.4)	54.3 (45.3–63.1)	15.7 (9.3–25.2)	
Place of Residence					<0.0001
Rural	3043 (78.9)	45.5 (42.9–48.1)	44.3 (42.1–46.5)	10.3 (9.0–11.8)	
Urban	2887 (21.1)	34.2 (31.4–37.1)	48.2 (45.5–51.0)	17.6 (15.4–20.2)	
Division of Residence					<0.0001
Dhaka Rural	669 (23.4)	39.2 (35.2–43.4)	46.3 (42.7–50.0)	14.5 (11.5–18.0)	
Barisal	729 (5.7)	40.4 (34.2–47.0)	45.0 (39.4–50.7)	14.6 (11.1–19.0)	
Chittagong	740 (19.9)	41.0 (35.8–46.4)	45.2 (40.8–49.8)	13.8 (10.8–17.4)	
Khulna	807 (12.3)	31.3 (26.8–36.2)	54.2 (49.5–58.8)	14.5 (11.9–17.5)	
Mymensingh	712 (8.6)	51.9 (46.4–57.3)	41.5 (36.8–46.4)	6.6 (4.4–9.7)	
Rajshahi	821 (13.8)	45.1 (37.6–52.8)	46.2 (39.4–53.2)	8.7 (6.5–11.5)	
Rangpur	777 (10.5)	60.0 (54.2–65.6)	34.2 (29.8–38.8)	5.8 (3.9–8.7)	
Sylhet	675 (5.7)	44.7 (39.5–49.9)	42.8 (37.9–47.9)	12.5 (10.0–15.5)	
Religion					0.3226
Islam	5121 (87.2)	43.7 (41.4–45.9)	44.7 (42.7–46.8)	11.6 (10.4–13.0)	
Other	809 (12.8)	39.1 (33.1–45.5)	47.5 (43.3–51.7)	13.4 (9.6–18.3)	

All the frequencies are unweighted frequencies, and percentages are weighted percentages. CI: Confidence Interval.

**Table 2:** Logistic regression showing the factors associated with the ideal cardiovascular metric in the adult Bangladeshi population.

Background Characteristics	COR (95% CI)	AOR (95% CI)
Age Group (in years)		
18–29	Ref	Ref
30–49	0.4 *** (0.4–0.5)	0.4 *** (0.4–0.5)
50–69	0.3 *** (0.2–0.3)	0.2 *** (0.2–0.3)
Sex		
Female	Ref	Ref
Male	0.7 *** (0.6–0.8)	0.7 *** (0.6–0.8)
Education		
No formal	Ref	Ref
Primary	0.9 (0.8–1.0)	0.7 *** (0.6–0.8)
Secondary	0.6 *** (0.5–0.7)	0.4 *** (0.4–0.5)
College and Higher	0.5 *** (0.4–0.6)	0.4 *** (0.3–0.5)
Marital Status		
Never married	Ref	Ref
Currently married	0.4 *** (0.4–0.5)	0.5 *** (0.4–0.7)
Divorced/widowed/separated	0.3 *** (0.2–0.4)	0.4 *** (0.3–0.6)
Place of Residence		
Rural	Ref	Ref
Urban	0.6 *** (0.5–0.6)	0.6 *** (0.6–0.7)
Division of Residence		
Dhaka Rural	Ref	Ref
Barisal	0.9 (0.8–1.2)	1.1 (0.9–1.4)
Chittagong	1.2 (0.9–1.5)	1.3 * (1.0–1.6)
Khulna	0.7 ** (0.6–0.9)	0.7 ** (0.6–0.9)
Mymensingh	1.4 ** (1.1–1.7)	1.6 *** (1.2–1.9)
Rajshahi	1.2 (1.0–1.5)	1.3 (1.0–1.6)
Rangpur	1.8 *** (1.5–2.3)	2.0 *** (1.6–2.5)
Sylhet	0.9 (0.7–1.1)	0.9 (0.7–1.1)
Religion		
Islam	Ref	Ref
Other	0.9 (0.7–1.0)	0.9 (0.8–1.1)

CI: Confidence Interval; COR: Crude Odds Ratio; AOR: Adjusted Odds Ratio. *** *p* < 0.001; ** *p* < 0.01; * *p* < 0.05.

## Data Availability

The dataset of WHO STEPS 2018 is available in the WHO NCD microdata repository. The data can be accessed from the following URL: https://extranet.who.int/ncdsmicrodata/index.php/home (accessed on 13 June 2021). Following the instruction of the WHO NCD microdata repository, the data can be downloaded.

## Supplementary Materials

The following supporting information can be downloaded at: https://www.mdpi.com/article/10.3390/epidemiologia3040040/s1, Table S1: Definition of ICH metrics. Table S2: Cross tabulation between the place of residence and division of residence (*N* = 5930).
