# Supplementary material for: Prevalence and Associated Factors with Ideal Cardiovascular Health Metrics in Bangladesh: Analysis of the Nationally Representative STEPS 2018 Survey
Source: Epidemiologia (Basel). 2022 Nov 30;3(4):533–43. doi: 10.3390/epidemiologia3040040 (PMC9778360; doi:10.3390/epidemiologia3040040)
Supplement: Supplementary file 1 [file epidemiologia-03-00040-s001.zip › epidemiologia-1954884-supplementary.pdf]

Table S1. Definition of ICH metrics.

**1.**	Ideal Blood Pressure (BP): Ideal BP was defined as SBP < 120 mmHg and DBP < 80 mmHg, and without taking any antihypertensive medication. Intermediate BP was defined as SBP 120–139 mmHg or DBP 80–89 mmHg or receiving any antihypertensive medication. Poor BP was defined as BP ≥ 140/≥ 90 mmHg.
**2.**	Ideal Total Cholesterol (TC): Ideal TC was defined as serum TC < 200 mg/dL and without taking any cholesterol-lowering medication. Intermediate TC was defined as serum TC 200–239 mg/dL or treated to TC < 200 mg/dL. Poor TC was defined as serum TC ≥ 240 mg/dL.
**3.**	Ideal Fasting Plasma Glucose (FPG): Ideal FPG was defined as <100 mg/dL and without any glucose-lowering medication. Intermediate FPG was defined as glucose 100–125 mg/dL or treated to <100 mg/dL. Poor FPG was defined as glucose ≥ 126 mg/dL.
**4.**	Body Mass Index (BMI): BMI was calculated by dividing the participant's weight measured in kilogram (kg), divided by height squared (m^2). Ideal BMI was defined as 18.5–24.9 kg/m^2. Intermediate BMI was defined as 25.0–29.9 kg/m^2. Poor BMI was defined as BMI ≥ 30 kg/m^2 [1].
**5.**	Smoking: Ideal smoking habit was defined as self-reported never smoking behavior. Intermediate smoking habit was defined as a former smoking habit. Poor smoking habit was defined as being a current smoker.
**6.**	Ideal diet: Daily intake of at least 4.5 servings of Fruits and vegetables was considered as an ideal diet. A poor diet was an intake of <4.5 servings per day.
**7.**	Physical activity: If a participant participated in at least 300 min of moderate-intensity aerobic physical activity/week or at least 150 min of vigorous-intensity aerobic physical activity/week, then it is defined as ideal physical activity. If a participant participated in at least 150 min of moderate-intensity aerobic physical activity/week or at least 75 min of vigorous-intensity aerobic physical activity/week, then it is defined as intermediate physical activity. Those who did not meet these criteria were categorized under poor physical activity.

[1] Underweight (BMI < 18.5 kg/m^2) was excluded from the analysis.

Table S2. Cross tabulation between the place of residence and division of residence (N = 5930).

**Division**	**Urban (*n*) ***	**Urban (%) ****	**Rural (*n*) ***	**Rural (%) ****	**Total**
Dhaka Rural	286	38.4	383	19.4	669
Barisal	364	4.9	365	6.0	729
Chittagong	375	18.0	365	20.5	740
Khulna	380	9.6	427	13.0	807
Mymensingh	340	5.7	372	9.4	712
Rajshahi	404	12.3	417	14.2	821
Rangpur	387	6.9	390	11.5	777
Sylhet	351	4.2	324	6.1	675
Total	2887		3043		5930

* Unweighted Frequency. ** Weighted Percentage.
